# Autophagy-Related Signature for Head and Neck Squamous Cell Carcinoma

**DOI:** 10.1155/2020/8899337

**Published:** 2020-10-19

**Authors:** Cheng Li, Zeng-hong Wu, Kun Yuan

**Affiliations:** ^1^Department of Otolaryngology Head and Neck Surgery, The Central Hospital of Wuhan, Tongji Medical College, Huazhong University of Science and Technology, Wuhan, Hubei, China; ^2^Department of Otolaryngology, Union Hospital, Tongji Medical College, Huazhong University of Science and Technology, Wuhan, Hubei, China; ^3^Department of Infectious Diseases, Union Hospital, Tongji Medical College, Huazhong University of Science and Technology, Wuhan 430022, China

## Abstract

**Background:**

Head and neck squamous cell carcinoma (HNSCC) is one of the most common malignancies in the world, with low survival and poor quality of life. Autophagy-associated genes (ATGs) have been reported to be involved in the initiation and progression of malignancies. Here, we aimed to investigate the association between autophagy-associated genes and the outcomes in HNSCC patients.

**Methods:**

We obtained ATGs with prognostic values by analyzing the datasets from The Cancer Genome Atlas (TCGA) and Human Autophagy Database (HADb). The enrichment functions of autophagy differential genes were analyzed by Gene Ontology (GO) and the Kyoto Encyclopedia of Genes and Genomes (KEGG). The Kaplan-Meier method was applied to the survival curve analysis. A prognostic autophagy-related gene signature was established, and its independence was verified.

**Results:**

We acquired a total of 529 samples and 232 ATGs; further, we identified 45 genes associated with prognosis and built a prognosis autophagy signature based on risk score of 15 genes. Patients were divided into two groups based on risk scores. The Kaplan-Meier curve illustrated that the survival rate of the high-risk group was significantly lower than that of the low-risk group in both the training group and validation group. The ROC curve revealed that the risk score had the highest AUC value in the 3rd and 5th years, reaching 0.703 and 0.724, which are higher than other risk factors such as gender, age, and TNM stage. The nomogram further confirmed its weight in the prognosis of HNSCC patients. Through KEGG and GO enrichment analyses, we observed that ATGs were involved in the tumorigenesis and invasion of tumor by various mediating pathways. We gained 3 hub genes (*MAP1LC3B*, *FADD*, and *LAMP1*) and further analyzed the survival curves, mutations, differential expressions, and their roles in tumors on the online websites.

**Conclusion:**

We identified a novel autophagy-related signature that may provide promising biomarker genes for the treatment and prognosis of HNSCC. We need to validate its prognostic value by applying it to the clinic.

## 1. Introduction

Head and neck tumors are common malignancies, of which more than 90% are head and neck squamous cell carcinoma (HNSCC). It mainly occurs in the lips, mouth, pharynx, throat, paranasal sinuses, and other head and neck areas [1]. The patients may present with nasal congestion, sore throat, oral ulcers, hoarseness, and local nodules. Some patients have metastases due to hidden lesions and inconspicuous symptoms. It is well known that smoking and alcohol abuse are typical high-risk factors for HNSCC. As people continue to research, we find that human papillomavirus (HPV) infection is also playing an increasingly important role in HNSCC [2]. The prevalence of HPV-related oropharyngeal cancer is rising especially in western countries [3, 4]. As the sixth most common tumor, HNSCC has an increasing annual incidence and a mortality rate of 40-50% [5]. Despite considerable progress in the treatment of HNSCC in recent years, its prognosis remains poor due to the lack of early diagnostic and predictive biomarkers [6, 7]. Thus, it is necessary to explore effective biomarkers for HNSCC treatment. At present, lncRNAs and miRNAs are believed to play an important role in tumorigenesis and have been identified as potential prognostic markers for HNSCC. Yang et al. [8] established an 8-lncRNA signature associated with the prognosis of HNSCC, and Hess et al. [9] identified prognostic 5-miRNA signature to independently predict disease control and survival in HNSCC patients. However, autophagy, as a crucial mechanism in tumorigenesis and progression, has not been studied on the effect of autophagy-associated genes on the prognosis of HNSCC.

Autophagy is the process of self-degrading damaged or degenerate proteins and organelles. Autophagy is thought to be related to malignant tumors [10], neurodegenerative diseases [11], immune diseases [12], infection [13], aging [14], and other diseases. Especially in tumors, it is a double-edged sword. Under physiological conditions, autophagy can prevent the accumulation of damaged substances and inhibit tumorigenesis. However, once a tumor has formed, cell autophagy can promote tumor growth. Autophagy promotes tumor growth, invasion, and metastasis in some cases [15, 16]. The formation and initiation of autophagosomes are complex processes, mainly the result of the coordination of three protein complexes. Activation of the *ULK1* complex triggers autophagy initiation [17–19] and regulates recruitment of a second kinase complex, the VPS34 complex. The third protein complex is composed of ATG16L1-ATG5-ATG12 conjugation machinery [20]. There are accumulating evidences that autophagy-mediated cell survival plays a role in the etiology and progression of HNSCC. Cigarette smoke exposure resulted in induction of autophagy by SIRT-1-PARP-1-dependent mechanism, leading to oncogenic mutations [21]. Inhibition of autophagy can dramatically enhance the infectivity of HPV-16 [22]. In addition, tobacco and alcohol have also been found to exert carcinogenicity by enhancing autophagy. Radiation resistance and chemoresistance result in poor therapeutic effect for many patients. Kuwahara et al. suggested that enhancement of autophagy is a potential modality for tumor refractory to radiotherapy [23]. Liu et al. found that autophagy inhibitor can enhance cisplatin-induced apoptosis in EC9706 cells, and it could be a promising strategy for the esophageal cancer [24].

Although some studies have explored the role of autophagy in the development of HNSCC, focusing on a single gene, however, little research has been done on the relationship between autophagy and the prognosis of HNSCC. In this study, we used the transcriptome data and corresponding clinical follow-up information to identify autophagy genes with significant prognostic value. Subsequently, we constructed a survival model to predict the prognosis of HNSCC.

## 2. Methods

### 2.1. Data Collection

TGGA, the largest cancer database with comprehensive cancer types and abundant clinical data, provides a fully shared interface for users to choose the required data. The RNA sequencing data for HNSCC patients were obtained from The Cancer Genome Atlas (TCGA; https://cancergenome.nih.gov); the clinical characteristics and survival information were also downloaded from here. After obtaining the data, we integrated the mRNA data with clinical information. The samples for which the gene expression was “zero” were excluded from the analysis. The list of ATGs got from Human Autophagy Database (HADb; http://www.autophagy.lu/), a web-based resource, provided a comprehensive and up-to-date list of human genes and proteins related to autophagy.

### 2.2. Identification of Differentially Expressed ARGS

We identified the differential genes in R package “limma” with the criteria of *P* < 0.05 and ∣logFC  | >1. Then, the expression levels in multiple samples and the differentially expressed ARGS were visualized by volcano plot and heat map.

### 2.3. Pathway Analysis

To analyze the potential function of differentially expressed ATGs, we used the “clusterProfiler” R package to perform the Gene Ontology (GO) and Kyoto Encyclopedia of Genes and Genomes (KEGG) analyses [25]. It was the process of classifying genes according to their functions. GO enrichment was carried out mainly from the following three levels: cellular components (CC), biological processes (BP), and molecular functions (MF), while KEGG analysis focused on metabolic pathways and molecular mechanisms.

### 2.4. Signature Establishment

Univariate Cox regression analysis and Lasso Cox regression analyses were carried out to evaluate the prognostic value of autophagy-related genes and establish the risk score [26]. Data with *P* value < 0.05 in univariate regression analysis will be further subjected to multivariate regression analysis. The prognostic gene signature was demonstrated as risk score = ∑_*i*=1_^ *n*^expr_genei_∗coefficient_genei_. The data would exclude the entire sample from the survey if any single value is missing to ensure data integrity and readability.

### 2.5. Statistical Analysis

The Kaplan-Meier curve was performed to describe the relationship between survival time and survival probability for high-risk group and low-risk group. The risk-related information is visualized through charts, including the following: the distribution of risk scores, risk-related survival status, and heat maps of prognostic ATGs. And the area under the curve (AUC) was created to predict prognostic value of the autophagy-related risk signature under the package of “survivalROC” [27]. And we use the “rms” R package to perform the nomogram [28]. The univariate and multivariate Cox regression analyses were carried out to verify the independence of signature; furthermore, we assessed the association between the signature and clinical parameters. We used Perl language for data matrix. All the statistical analyses of this research were conducted using the R software (version 3.6.3). Data with *P* < 0.05 were considered statistically significant.

### 2.6. Online Database Analysis

The PPI network information of prognostic-related autophagy genes was obtained from the STRING (https://string-db.org/) online database. Then, the most significant modules in the PPI networks were identified from the plug-in Molecular Complex Detection (MCODE) of Cytoscape (version 3.6.0). After screening the hub genes, we explored their relevant information and functions, including genome mutations, tumor immune microenvironments, and survival status from the online website. Genomic mutations were analyzed from cBioPortal for Cancer Genomics (http://www.cbioportal.org/), a web resource for exploring, visualizing, and analyzing multidimensional cancer genome data. Tumor Immune Estimation Resource (TIMER; http://cistrome.shinyapps.io/timer), a user-friendly web interface, provided us for comprehensively investigating molecular characterization of tumor-immune interactions. We used the Kaplan-Meier plotter online tool to plot survival curves and perform the survival analysis.

## 3. Results

### 3.1. Construction and Assessment of the ATG Prognostic Signature

A total of 529 samples containing transcriptome and clinical data were obtained from the TCGA-HNSC database, including 487 HNSCC samples and 42 normal samples. 232 autophagy-related genes were downloaded from the HADb database. Subsequently, a total of 45 prognostic-related autophagy genes were selected for further evaluation based on *P* < 0.05. Then, we utilized the LASSO Cox regression model to screen out 15 autophagy genes with the most prognostic value. The risk score was calculated based on their expression level and associated Cox regression coefficient. The risk score = (expr EEF2K∗−0.290) + (expr LAMP1∗0.187) + (expr GABARAPL2∗0.317) + (expr MAP1LC3A∗−0.239) + (expr WIPI2∗0.590) + (expr IKBKB∗−0.309) + (expr ST13∗0.380) + (expr NAMPT∗0.123) + (expr MAP2K7∗−0.575) + (expr GAPDH∗0.270) + (expr ATIC∗0.248) + (expr VAMP7∗0.370) + (expr SAR1A∗0.493) + (expr NKX2 − 3∗−0.278) + (expr TSC2∗0.307).

**Figure 1:**
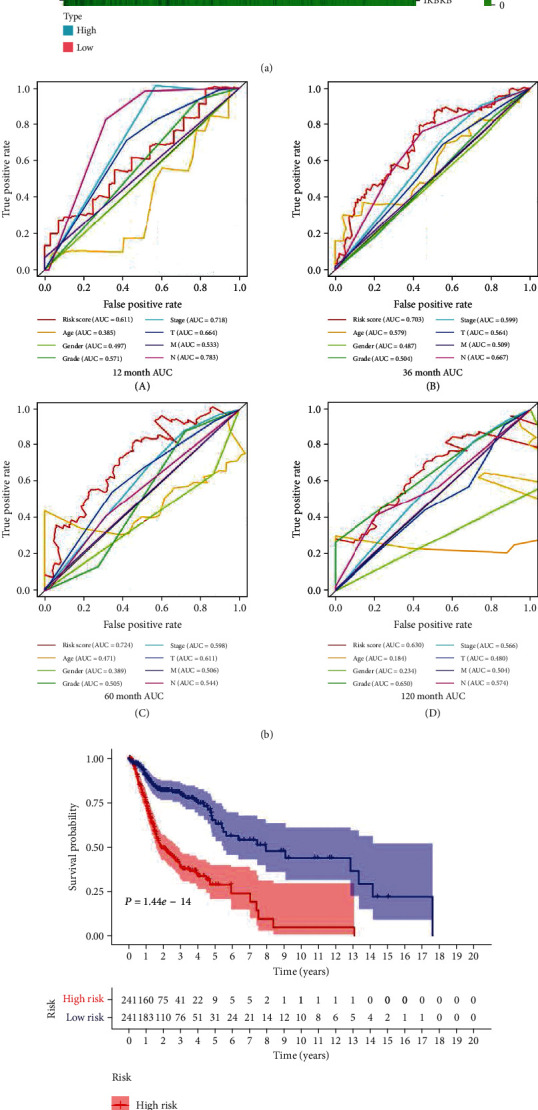
(a) The risk score increased from green to red; green and red scatter represent alive and dead, respectively, in the survival time plot; heat map of the 15 autophagy-related genes. (b) The ROC curves of OS for autophagy-related signature score, age, gender, grade, stage, T, M, and N in the 1st, 3rd, 5th, 10th years. (c, d) The Kaplan-Meier curve of OS: HNSCC patients in the high-risk group had worse outcomes than those in the low-risk group in both the training group (*P* < 0.01) (c) and validation group (*P* = 0.03224) (d).

**Figure 2:**
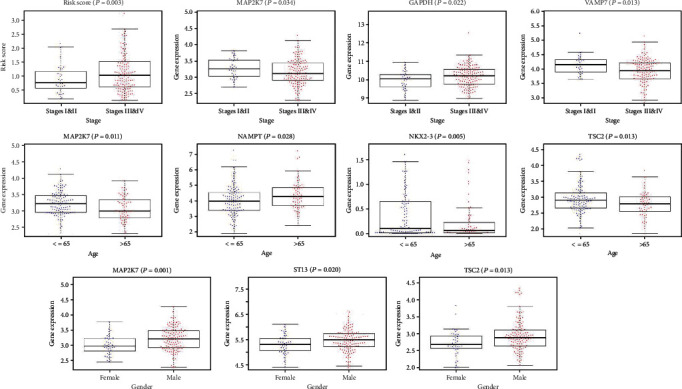
Association between the autophagy-related signature and clinical parameters.

Patients were divided into high-risk and low-risk groups with the median risk score as cutoff value (Figure 1(a)). The Kaplan-Meier log-rank test revealed a significant difference in the overall survival (OS) rate between two groups, that is, the prognosis of the high-risk group is significantly worse than that of the low-risk group in both the training group (Figure 1(c)) and validation group (Figure 1(d)). The distribution of risk score, survival time, and gene expression in patients at different risk was visually shown in Figure 1(a). As the patient's risk increased, the survival time decreased and the deaths increased. The ROC curve revealed that the risk score had the highest AUC value in the 3rd and 5th years, reaching 0.703 and 0.724, which were higher than other risk factors such as gender, age, and TNM stage (Figure 1(b)). The univariate and multivariate Cox regression analyses confirmed the independence of the model (Table 1). The relationship between risk model and clinical parameters was also shown in the plot (Figure 2).

### 3.2. Constructing a Predictive Nomogram

**Figure 3:**
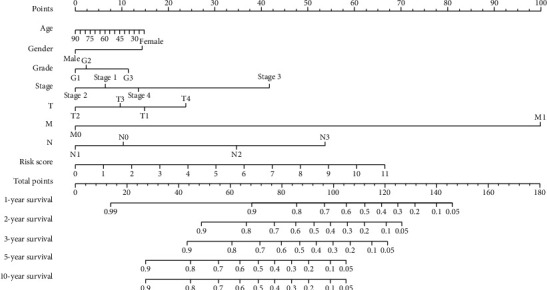
A nomogram for predicting 1-, 2-, 3-, 5-, and 10-year survival rate of HNSCC patients was established.

On the basis of LASSO logistic regression algorithm, nomogram indicated the survival rate of HNSCC individually, that was, using the prognostic model and multiple clinical indicators to predict certain clinical outcomes. The nomogram ultimately included 7 clinical variables: age, gender, grade, stage, T, M, and N (Figure 3). We may get more beneficial prognostic value through integrating our signature with clinical characteristics.

### 3.3. Function Enrichment Analysis

**Figure 4:**
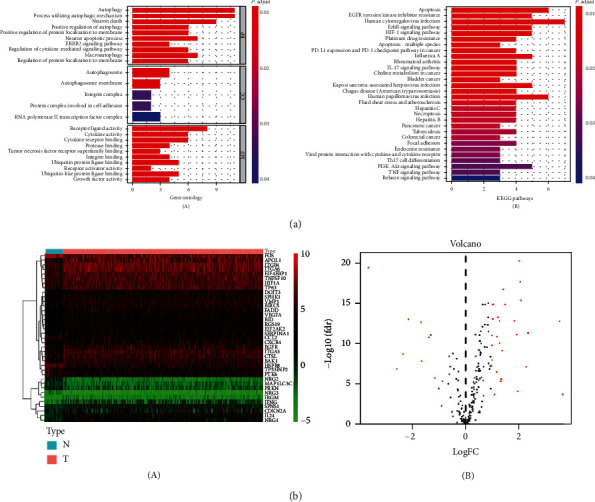
(a) Gene functional enrichment of differentially expressed ATGs: GO analysis (A) and KEGG pathways analysis (B). (b) Differential expression of autophagy-related genes was visualized in the heat map (A) and the volcano plot (B).

We discussed potential signaling pathways' 37 differential ATGs. Gene Ontology made simple annotations on gene functions, participating biological pathways, and localization in cells (Figure 4(a)). The biological processes were mainly involved in the autophagic mechanism, apoptosis, regulation of protein localization to membrane, cytokine activity, tumor necrosis factor, and ubiquitin protein ligase. Gene products were mainly localized in autophagosome, autophagosome membrane, integrin complex, and protein complex involved in cell adhesion. Their functions mainly included the following: receptor ligand activity, cytokine activity, and cytokine receptor binding. KEGG analysis indicated that ATGs were mainly related to the following pathways, such as EGFR tyrosine kinase inhibitor resistance, human cytomegalovirus infection, PD-1 checkpoint pathway in cancer, and HIF-1 signaling pathway (Figure 4(a)). The heat map intuitively shows the expression levels of differential autophagy genes in different samples (Figure 4(b), A). The logFC and -log10 of FDR were visualized in the volcano plot (Figure 4(b), B).

### 3.4. Online Database Analysis

**Figure 5:**
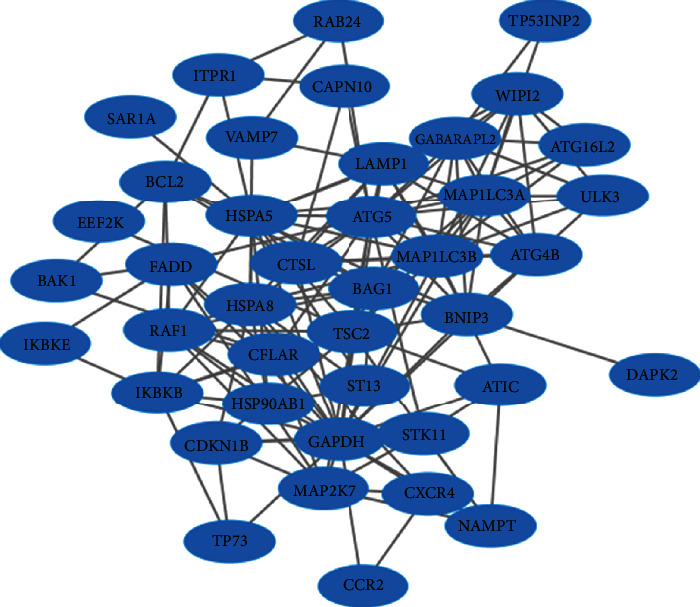
Network of prognostic autophagy genes is visualized by the Cytoscape 3.6.0 software.

**Figure 6:**
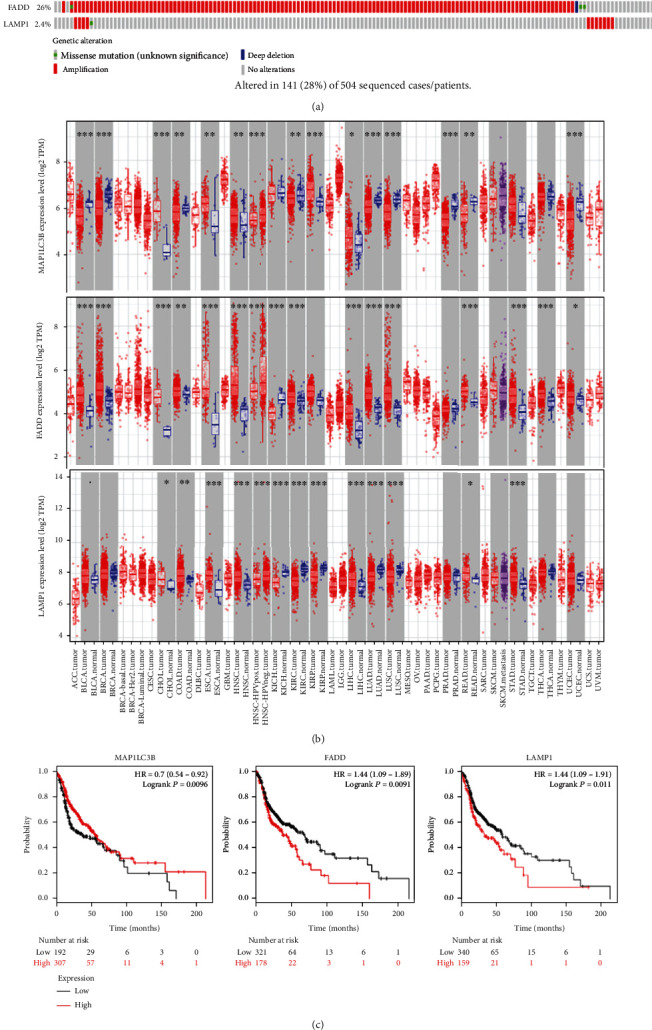
(a) The types, ratios, and distributions of *MAP1LC3B*, *FADD*, and *LAMP1* mutations are shown on OncoPrint. (b) Differential expression of *MAP1LC3B*, *FADD*, and *LAMP1* between tumors and normal tissues. *P* value significant codes: 0 ≤  ^∗∗∗^ < 0.001 ≤  ^∗∗^ < 0.01 ≤  ^∗^ < 0.05. (c) The Kaplan-Meier curves of *MAP1LC3B*, *FADD*, and *LAMP1* in HNSCC patients.

**Figure 7:**
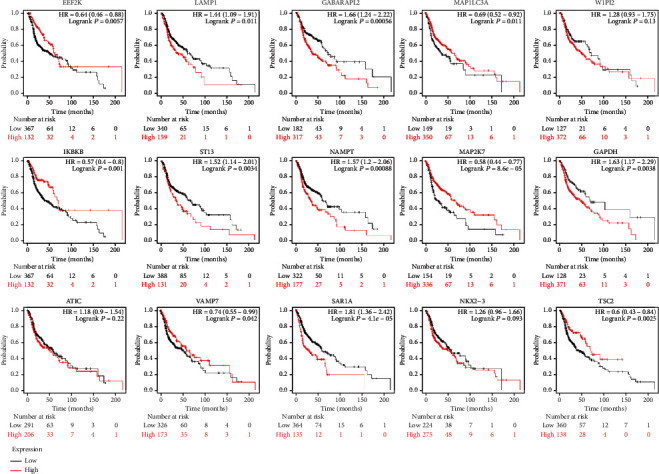
The single prognostic KM curves of 15 autophagy-related genes have been obtained, which were significant for the prognosis of HNSCC (*P* < 0.05) except WIPI2, ATIC, and NKX2-3.

45 prognostic-related autophagy genes were linked and formed a tight protein-protein interaction network (Figure 5). And a total of 3 genes (*MAP1LC3B*, *FADD*, and *LAMP1*) were identified as hub genes by the module of MCODE of Cytoscape. Subsequently, we used multidimensional survey ways to explore the hub genes based on Online database analysis. The mutations of the hub genes were shown in Figure 6(a). We concluded that the three genes showed significant differential expression in a variety of tumors, including HNSCC tumors (Figure 6(b)). Kaplan-Meier survival curves of 3 hub genes indicated the significant differences in survival (Figure 6(c)). The single KM curves of 15 prognostic autophagy-related genes were also obtained from Kaplan-Meier plotter, as shown in Figure 7. We can see that almost all genes except WIPI2, ATIC, and NKX2-3 are significant for the prognosis of HNSCC (*P* < 0.05).

## 4. Discussion

As is known to all, the treatment of HNSCC is mainly combined surgery, chemotherapy, and radiotherapy. Early stage tumors (stages I and II) may achieve satisfactory results by surgery, while for advanced tumors (stages III and IV) or recurrent tumors, chemotherapy or radiotherapy is mainly adopted. Although current treatment technologies are constantly improving, therapeutic resistance, such as radioresistance and chemoresistance are still the key factors for poor prognosis of HNSCC. Current research suggests that the autophagy genes may contribute to the carcinogenicity of smoking, drinking, and HPV infection, as well as to the process of chemoresistance and radiation resistance [29]. We deem that the autophagy genes may be important in opening up potential clinical applications and in the proper assessment of prognosis. Therefore, we here studied the pathway function of autophagy gene, screened the prognostic autophagy gene in combination with clinicopathological conditions, and established a reliable prognostic signature.

In this study, we conducted GO and KEGG enrichment analysis of 37 autophagy-related differential genes to further analyze their functions and pathways in tumors. In this result, a variety of tumor-related signals were presented, such as regulation of autophagy, apoptosis, regulation of protein localization to membrane, cytokine activity, tumor necrosis factor, and ubiquitin protein ligase. The formation of autophagy is a process involving multiple genes and related proteins. Autophagy is critical in maintaining cell homeostasis to play a tumor suppressive role, but can also promote tumor progression in tumor cells. Autophagy is involved in removing dysfunctional mitochondria [30] and can also mediate anti-inflammatory effects [31], which are associated to some extent with malignant transformation. The dysregulation of ubiquitin ligases is related to a variety of cellular processes, directly involved in human malignancies [32, 33]. Membrane proteins, as anchors on cell surface, play a key role in signal transduction. Some studies have observed elevated levels of some cytokines in HNSCC patients, as well as decreased levels of others [34]. The pathway analysis of autophagy gene concluding EGFR tyrosine kinase inhibitor resistance, human cytomegalovirus infection, PD-1 checkpoint pathway in cancer, and HIF-1 signaling pathway further confirmed the correlation between autophagy gene and malignant tumor. EGFR, also known as ErB1, is one of the epidermal growth factor receptors (HER). The overexpression of EFGR is considered to be the pivotal transforming events of the HNSCC [35]. Inhibition of EFGR expression as a means of disease treatment, however, resistance to EGFR tyrosine kinase inhibitors often bring about unsatisfactory results. There has been evidence that CMV may induce salivary gland tumors in addition to being closely related to the genitourinary and nervous systems [36]. Philip et al. found that activation of HIF-1 could induce autophagy and block apoptosis, so that malignant cells could continue to survive and maintain the invasion characteristics [37]. At the same time, the expression of HPV-16 protein can increase the accumulation of HIF-1 [38]. In addition, other pathways such as PD-L1 expression and PD-1 checkpoint pathway, the IL-17 signaling pathway, platinum drug resistance, and the mediation of multiple viral infections have also been found to be involved in the growth, invasion, and metastasis of tumor cells [39–41].

A total of 45 autophagy genes with prognostic values were screened out. These genes were made into PPI network diagram and further screened out 3 hub genes with high connectivity degree, namely, *MAP1LC3B*, *FADD*, and *LAMP1*. Microtubule-associated protein-1 light chain 3 beta (*MAP1LC3B*) is significantly associated with adverse clinicopathological outcomes in some cancer types. Liu et al. found that the expression levels of *MAP1LC3B* and *SQSTM1* in tumor tissues were higher than those in adjacent normal tissues, suggesting that MAP1LC3B promoted the tumorigenesis and drug resistance of oral squamous cell carcinoma [42]. However, contrary to the above, high LC3 expression appears to be associated with reduced non-small-cell lung cancer invasiveness [43]. Fas-associated death domain (*FADD*) mediates multiple death receptor-induced apoptotic signaling pathways and also plays a role in T cell proliferation and embryonic development. Its effect on tumor prognosis depends on the tumor type and cell environment, for example, it is a poor prognostic marker in head and neck tumor, lung cancer, and cervical cancer, while it is beneficial for thyroid cancer [44]. Lysosome-associated membrane protein-1 (*LAMP1*) is a member of the lysosomal membrane protein involved in the induction of cell death. Data showed that *LAMP1* was highly expressed in a variety of tumors, such as laryngeal cancer [45], ovarian cancer [46], and breast cancer [47].

The OncoPrint tab summarized genomic changes in three genes in the sample set, whose distributions in the sample were almost mutually exclusive. *FADD* mutation rate was the highest, up to 26%, mainly manifested by amplification. Major amplification and a small amount of missense mutation were found to occur in *LAMP*, and *MAP1LC3B* mutation was the least (0.8%), with deep deletion and amplification. The results of Kaplan-Meier curves for the 3 hub genes were statistically significant. As the results showed, in patients with HNSCC tumors, high *FADD* and *LAMP* expression levels had lower survival rates, while high *MAP1LC3B* levels were associated with favorable prognosis. We obtained the data of gene differential expression from the TIMER website. The three genes showed significant differential expression in a variety of tumors, and they were highly expressed in HNSCC patients and poorly expressed in normal samples. In any case, three autophagy genes were differentially expressed in a variety of tumors, which has been reported in previous studies. However, whether they have prognostic value in HNSCC requires further study.

In some cases, the prognostic accuracy of gene markers is better than TNM staging. We obtained 15 genes with prognostic value by optimizing models and calculated the risk score based on the gene expression and risk coefficient of each gene, so as to accomplish risk stratification. The Kaplan-Meier curve displayed the survival rate of the high-risk group was significantly better than that of the low-risk group, especially in the 3rd, 5th, and 10th years. Just as shown in the ROC curve, the AUC of risk score in the third and fifth years was greater than that of age, gender, and traditional TNM staging under the same conditions, which confirmed the stability and applicability of risk signature in the survival prognostic ability of HNSCC. And multivariate analysis showed that risk score was an independent prognostic factor for HNSCC patients. We further developed the nomogram which integrated risk score with various clinical parameters to more intuitively highlight their weight in the prognosis. Risk score was more weighted than clinical traits other than M staging. It also indicated the significance of our prognostic model. In addition, clinical correlation analysis revealed that risk increased significantly as tumor stage increased. The expression levels of *ATIC*, *GAPDH* and *MAP1LC3A*, *MAP2K7*, and *NAMPT* are associated with TNM staging, while NAMPT and *NKX2-3* may be relevant to the gender.

## 5. Conclusions

In summary, although there have been previous studies on the relationship between autophagy genes and tumors, to our knowledge, this is the first study to explore the correlation between autophagy genes and the outcomes of HNSCC patients. However, this study still has some limitations. Firstly, our data was retrospective, which should be verified in the future. Secondly, we only focused on autophagy genes, and the result could not represent all gene spectrum. In this study, we got the 15 autophagy genes with significant prognostic values. Further, we established the autophagy-related prognostic signature and confirmed its independence. At the same time, we performed the functional analyses of autophagy genes. Our signature may provide promising biomarker genes for the treatment and prognosis of HNSCC. We need to apply the autophagy-related signature to clinical practice in order to validate its prognostic value.

## Figures and Tables

**Table 1:** Univariate and multivariate analyses of overall survival.

Items	UniCox				MultiCox			
	HR	HR 95L	HR 95H	*P* value	HR	HR 95L	HR 95H	*P* value
Age	1.013	0.987	1.040	0.325	1.016	0.987	1.047	0.276
Gender	0.784	0.405	1.517	0.469	0.721	0.348	1.493	0.379
Grade	1.183	0.731	1.915	0.494	1.074	0.623	1.852	0.798
Stage	1.812	1.110	2.959	0.017	1.152	0.552	2.405	0.706
T	1.377	0.990	1.916	0.058	1.178	0.739	1.876	0.491
M	144.458	9.034	2309.987	<0.001	191.648	10.634	3454.067	<0.001
N	1.593	1.162	2.185	0.004	1.378	0.918	2.070	0.122
Risk score	1.445	1.223	1.707	<0.001	1.445	1.203	1.735	<0.001

## Data Availability

Data sharing is not applicable to this article as no datasets were generated or analyzed during the current study.

## Abbreviations

HNSCC: Head and neck squamous cell carcinoma
ATG: Autophagy-associated gene
TCGA: The Cancer Genome Atlas
HADb: Human Autophagy Database
GO: Gene Ontology
KEGG: The Kyoto Encyclopedia of Genes and Genomes
AUC: Area under the curve.
